# Overexpression of CHAF1A is associated with poor prognosis, tumor immunosuppressive microenvironment and treatment resistance

**DOI:** 10.3389/fgene.2023.1108004

**Published:** 2023-03-09

**Authors:** Xia Sun, Qiushuang Ma, Yahong Cheng, Huangwei Huang, Jing Qin, Mengchen Zhang, Sifeng Qu

**Affiliations:** ^1^ Department of Pharmacology, School of Basic Medical Sciences, Cheeloo College of Medicine, Shandong University, Jinan, Shandong, China; ^2^ Medical Integration and Practice Center, Cheeloo College of Medicine, Shandong University, Jinan, Shandong, China; ^3^ Department of Urology, Qilu Hospital of Shandong University, Shandong University, Jinan, Shandong, China; ^4^ Institute of Materia Medica, Shandong First Medical University & Shandong Academy of Medical Sciences, Jinan, Shandong, China

**Keywords:** CHAF1A, cancer, prognosis, metabolism, immune

## Abstract

**Background:** As distinct marker of proliferating cells, chromatin assembly factor-1 (CAF-1) was critical in DNA replication. However, there is paucity information about the clinical significance, functions and co-expressed gene network of CHAF1A, the major subunit in CAF-1, in cancer.

**Methods:** Bioinformatic analysis of CHAF1A and its co-expression gene network were performed using various public databases. Functional validation of CHAF1A was applied in breast cancer.

**Results:** Overexpression of CHAF1A was found in 20 types of cancer tissues. Elevated expression of CHAF1A was positively correlated with breast cancer progression and poor patients’ outcome. The analysis of co-expression gene network demonstrated CHAF1A was associated with not only cell proliferation, DNA repair, apoptosis, but cancer metabolism, immune system, and drug resistance. More importantly, higher expression of CHAF1A was positively correlated with immunosuppressive microenvironment and resistance to endocrine therapy and chemotherapy. Elevated expression of CHAF1A was confirmed in breast cancer tissues. Silencing of CHAF1A can significantly inhibit cell proliferation in MDA-MB-231 cells.

**Conclusion:** The current work suggested that overexpression of CHAF1A can be used as diagnostic and poor prognostic biomarker of breast cancer. Higher expression of CHAF1A induced fast resistance to endocrine therapy and chemotherapy, it may be a promising therapeutic target and a biomarker to predict the sensitivity of immunotherapy in breast cancer.

## Introduction

Cancer is the leading cause of death which is a major threaten to public health worldwide (Sung et al., 2021). Cancer cell proliferation is the fundamental precondition for disease development and progression. Among the proteins involved in the DNA assembly into chromatin, the chromatin assembly factor-1 (CAF-1) plays important role to promote assembly of chromatin and histone proteins deposition on to the DNA (Shen et al., 2020). CAF-1, an outstanding marker of proliferating cell (Polo et al., 2004), has been found to be the key regulator in DNA replication and chromatin restoration (Zheng et al., 2018). The expression of CAF-1 is positively correlated with the expression of Ki-67 in cancers (Sykaras et al., 2021).

CAF-1 is a nuclear complex containing three subunits in human cells, CHAF1A (p150), CHAF1B (p60), and RBBP4 (p48) (Sykaras et al., 2021). Among the three subunits, CHAF1A is the major one and plays essential role in CAF-1 complex (Liu et al., 2017; Tao et al., 2021). It is shown that cells were not in S phase when Chaf1a failed to bind to mouse heterochromatin-binding protein-1 (Hp1) during mitosis (Murzina et al., 1999). However, homozygous deletion of Chaf1a in mice was fatal to mice embryos. And absence of Chaf1a in these embryos led to changes of the nuclear organization in constitutive heterochromatin (Houlard et al., 2006). In recent years, studies have suggested that the elevated expression of CHAF1A is closely correlated with the development of some types of cancer, such as neuroblastoma, lung cancer, ovarian cancer, and gastric cancer (Barbieri et al., 2014; Liu et al., 2017; Xia et al., 2017; Zheng et al., 2018). Therefore, CHAF1A is considered to be one of the important oncogenic factors. However, CHAF1A has rarely been reported in breast cancer. More importantly, the precise mechanism of breast cancer development and progression remained unclear. Therefore, it was crucial to find a novel and reliable biomarker for diagnosis, prognosis and prediction of treatment response in breast cancer.

In our study, we took a comprehensive approach to investigate the genomic alterations of CHAF1A and demonstrate CHAF1A expression profiles in various cancer types. Our study not only confirmed CHAF1A abnormally high expression in cancers especially in breast cancer, but also demonstrated a strong correlation between CHAF1A overexpression, breast cancer molecular subtype, prognosis and treatment response. Co-expression network analysis was conducted for further investigation of the underlying roles of CHAF1A. Tumor immunosuppressive microenvironment was also explored to find out the association between CHAF1A and immune cells. Here, we provide evidence to demonstrate that CHAF1A could be served as a promising biomarker for breast cancer diagnosis, prognosis, sensitivity of immunotherapy and target of therapeutics.

## Materials and methods

### COSMIC (catalogue of somatic mutations in cancer) database analysis

The COSMIC database (Tate et al., 2019) is a comprehensive platform to explore somatic mutations in human cancers. The latest version was released on 28 May 2021 (v94), which included gene mutations, copy number variations, genomic rearrangements and gene fusions across 1,491,089 cancer samples. As such, genomic alterations of CHAF1A were summarized using COSMIC database.

### Assessment of CHAF1A expression from Oncomine database

Oncomine (Rhodes et al., 2007) is a cancer database with genome-wide expression analyses of 715 datasets and a total of 12764 normal and 86733 tumor samples. Through “differential analysis” module, the expression of a single gene could be analyzed across various cancer types compared with corresponding normal samples.

### TIMER 2.0

TIMER 2.0 (Li et al., 2016; Li et al., 2017; Li et al., 2020) is a database for comprehensive investigation of immune cells infiltrated in cancer tissues in a large variety of malignant diseases. There are three major modules for analysis of cancer exploration, immune association and immune estimation, including gene expression, gene correlation, immune infiltration in this study.

### UALCAN

UALCAN (Chandrashekar et al., 2017; Chen et al., 2019; Chen et al., 2022) is an on-lined data-mining resource to analyze gene expression and protein expression profile across various cancer types based on publicly available cancer OMICS data, including TCGA, CBTTC and CPTAC. It provides patient survival information for lincRNA-coding, miRNA-coding and protein-coding genes at the same time, which could discover candidate proteins that may be used as tumor biomarkers.

### Kaplan-Meier Plotter analysis

The Kaplan-Meier plotter (Győrffy, 2021; Lánczky and Győrffy, 2021) is an on-line platform which contains the expression of 30,000 genes and the survival data over 25,000 patients from 21 cancer types. The correlation of CHAF1A expression (Jetset Best Probe: 214426_x_at) and patients’ survival were investigated by applying a log-rank test.

### cBioPortal (cBio cancer genomics portal) analysis

The cBioPortal (Cerami et al., 2012; Gao et al., 2013) is an open source which provides a comprehensive platform for exploration, interactive visualization and analysis of large-scale cancer genomic datasets for scientific research. The co-expression data of CHAF1A were downloaded in cBioportal database, which was used for further investigation.

### OmicShare online platform

OmicShare (Su et al., 2019; Wang et al., 2020) is a platform for comprehensive data analysis. It contains multiple modules for different uses, such as heatmap, Gene Ontology (GO) enrichment, senior bubble plot, pathway enrichment and so on.

### GSEA (gene set enrichment analysis)

GSEA (Mootha et al., 2003; Subramanian et al., 2005) is a bioinformatic software that analyze and determine the statistically significant and concordant differences between two datasets based on *a priori* defined set of genes. The enrichment of CHAF1A co-expression genes were used to investigate potential functions and mechanisms of CHAF1A. Normalized enrichment score (NES) was calculated, and nominal *p*-value <0.05 was considered as statistically significant enrichment terms.

### GEPIA (gene expression profiling interactive analysis)

GEPIA (Tang et al., 2017) is a platform containing 9,736 tumors samples and 8,587 normal samples based on TCGA and Genotype-Tissue Expression (GTEx) projects. It is applied for the pan-cancer analysis of the RNA-sequencing expression data, including gene expression, gene correlation, survival rate and so on.

### Immunohistochemistry (IHC) and western blot

Breast cancer tissues and adjacent benign tissues were obtained in Qilu Hospital of Shandong University. The study was approved by the Ethics Committee of School of Basic Medical Sciences of Shandong University. Preparation of paraffin-embedded tissue sections, immunohistochemical and western blot analyses were performed as previously reported (Qu et al., 2018). The anti-human CHAF1A antibody (ab126625, Abcam, Cambridge, UK) was used to detect the expression of CHAF1A.

### Cell culture and shRNA transfection

MDA-MB-231 cells were obtained from the Cell Bank of the Chinese Academy of Sciences (Shanghai, China). Cells were cultured with Dulbecco’s Modified Eagle’s Medium (DMEM, CM15019, Macgene, China) with 10% FBS (S711-001S, Lonsera, Uruguay). Cells were maintained as monolayer cultures at 37°C in a humidified incubator with no CO_2_ atmosphere. shRNA of CHAF1A was obtained from GenePharma (Shanghai, China). Cells were transfected with shRNA using polybrene (GenePharma, Shanghai, China) according to the manufacturer’s guidelines.

### CCK8 analysis

The CCK8 Cell Counting Kit (Vazyme, Biotech Co., Ltd) assay was performed using the protocol reported (Qin et al., 2021). MDA-MB-231 cells with stable silencing of CHAF1A using shRNA were seeded in 96-well plates. After replaced with fresh culture medium, 10 μL CCK-8 solution was added to each well and incubated at 37 °C for 3 h. The absorbance was determined at 450 nm on microplate absorbance reader (Bio-rad, United States) at 0h, 48h, and 96 h, respectively.

### Statistical analysis

All of these analyses were taken with *p* < 0.05 as the significance threshold, unless specificlly mentioned. ANOVA was applied to study the expression of CHAF1A in GEPIA database. Log-rank *p*-value was used for Kaplan-Meier Plotter analysis, and nominal *p*-value was used for GSEA analysis. Students’ t-test was used to analyze the data in Oncomine, UALCAN and CCK8. Spearman’s Correlation was used for selection of co-expression genes in cBioportal database and TIMER database. A statistic significant correlation between candidate gene(s) and immune cell(s) was considered if |Rho| > 0.1.

## Results

### Genomic alterations of CHAF1A

In order to identify the contribution of CHAF1A gene in human cancers, COSMIC (v94 GRCh38) (Tate et al., 2019) was applied for genomic alteration assessment. In the latest released version of COSMIC, CHAF1A was tested in 39,615 cancer samples across 40 different types of cancer. The total mutation frequency of CHAF1A was 1.105%, while missense mutation counted for 0.792% and the rest were nonsense and synonymous mutations (Table 1). Compared with mutation, Insertion and Deletion were rarely found in cancers. Only one Frameshift Insertion (0.003%), three Inframe Deletion (0.008%) and five Frameshift Deletion (0.012%) were found in cancers (Table 1). The total frequency of copy number alterations was 0.089% in cancers, including 0.008% for copy number gain while 0.081% for copy number loss (Table 1). As shown in Supplementary Table S1, one copy number gain was found in malignancies of central nervous system, haematopoietic and lymphoid, and lung, respectively. Copy number loss was found in 12 malignancies, with the highest rate of 1.154% in upper aerodigestive tract. Taken together, the data indicated that no major alterations in either sequence or copy number of the CHAF1A gene were responsible for cancer development.

**TABLE 1:** Genetic alterations affecting *CHAF1A* in 39,615 cancer samples (COSMIC database).

Genetic alteration	Number	Percentage
Substitution Nonsense	14	0.035%
Substitution Missense	314	0.792%
Substitution Synonymous	110	0.277%
Inframe Insertion	0	0
Frameshift Insertion	1	0.003%
Inframe Deletion	3	0.008%
Frameshift Deletion	5	0.012%
Complex Mutation	0	0
Others	14	0.035%
Copy Number Gain	3	0.008%
Copy Number Loss	32	0.081%

### Overexpression of CHAF1A in human cancers

**FIGURE 1:**
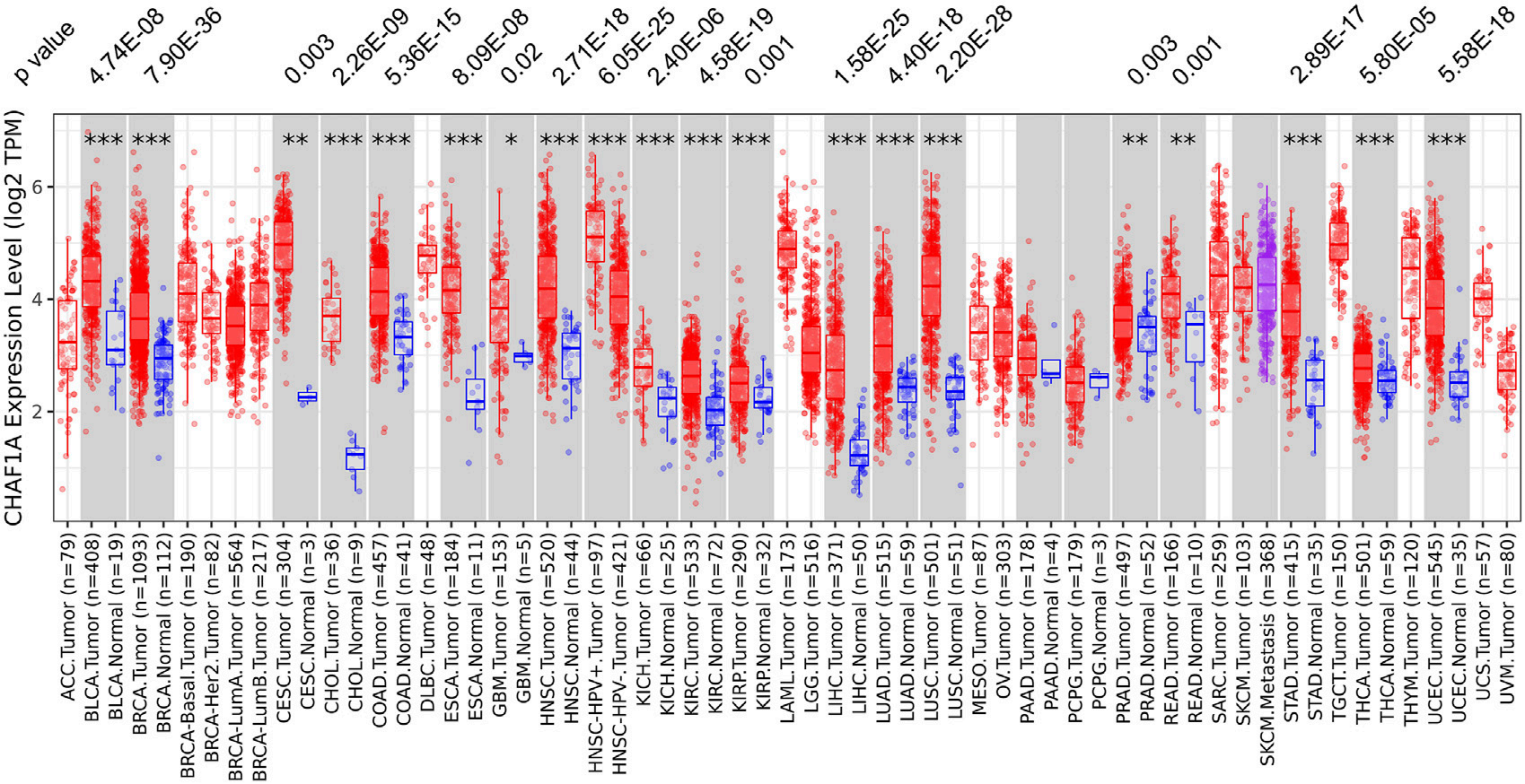
The expression of CHAF1A is significantly higher in malignant tissues compared with normal tissues in various cancer types. In TIMER 2.0 database, CHAF1A is significantly highly expressed in 20 types of malignancies compared with normal tissues. The statistical significance was annotated by stars (*: *p* < 0.05; **: *p* < 0.01; ***: *p* < 0.001).

Using TIMER 2.0 database (Li et al., 2017; Li et al., 2020), the gene expression of CHAF1A was investigated in various cancer types. Among the 22 paired tumor and corresponding normal samples, the expression of CHAF1A was significantly elevated in 20 types of malignancies compared with normal tissues (Figure 1). In consistent with the result from TIMER 2.0 database, it was also found abnormally high expression of CHAF1A in 36 study cohorts covering 17 types of cancers in Oncomine database (Rhodes et al., 2007) (Supplementary Table S2). In both two databases, breast cancer cohort contains the greatest amount of patient samples. The patient number included in breast cancer cohorts is more than other types of cancer, and with statistical significance as shown in Figure 1 and Supplementary Table S2. In addition, breast cancer is a leading cause of death in female. Thereby, we focused on breast cancer in the further investigation.

### Correlation of CHAF1A expression with breast cancer phenotype and prognosis

**FIGURE 2:**
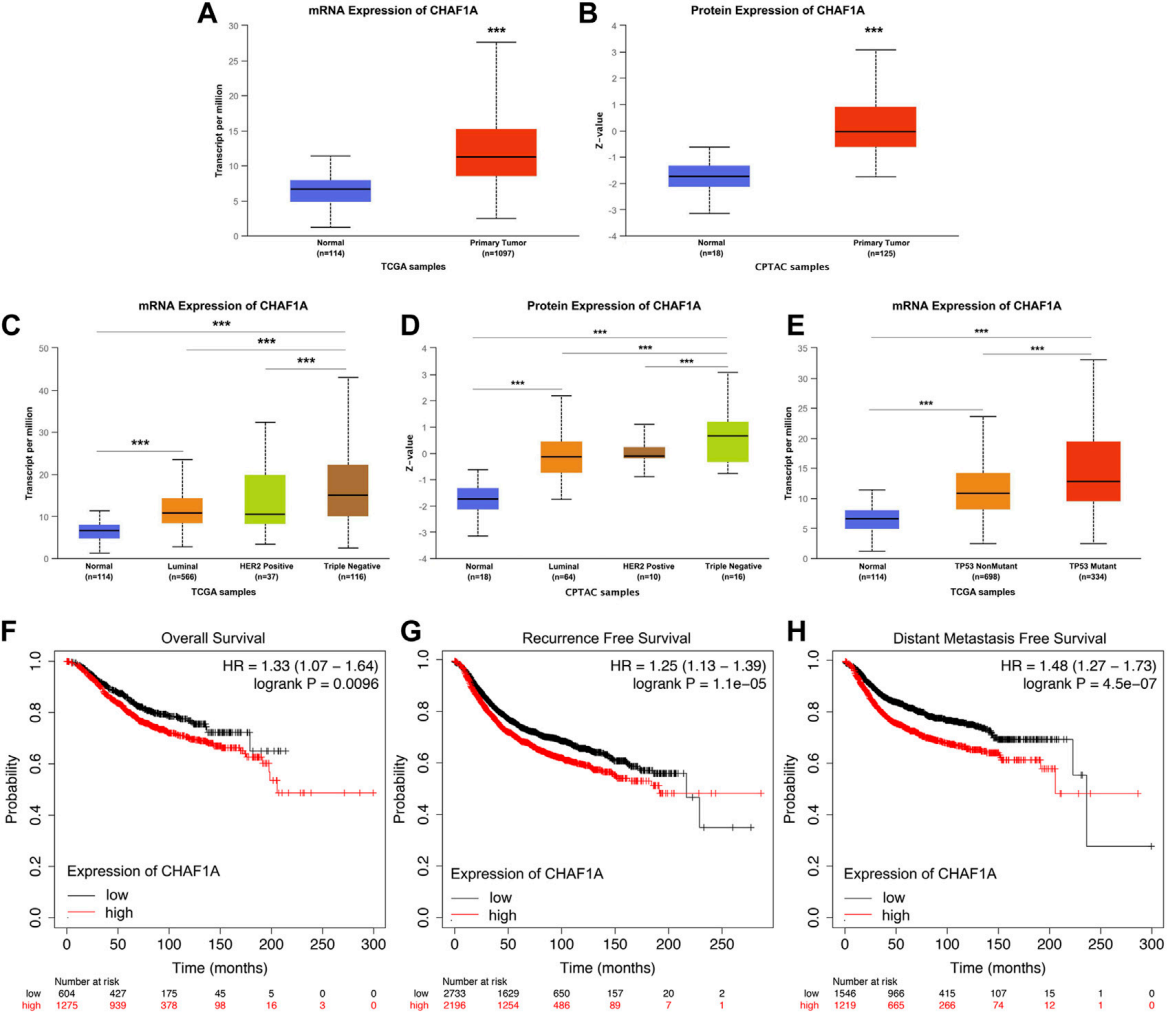
Elevated expression of CHAF1A in breast cancer tissues was significantly correlated with breast cancer patients’ poor prognosis. By applying UALCAN database **(A,B)** Both mRNA expression and protein expression of CHAF1A were dramatically increased in breast cancer tissues. **(C,D)** CHAF1A was overexpressed in all subtypes of breast cancer samples compared with normal ones. And compared with either Luminal or HER2 positive breast cancers, CHAF1A showed the highest expression in TNBC. **(E)** The expression of CHAF1A was higher in TP53 mutant breast cancer tissues compared to either TP53 wild type or normal tissues (***: *p* < 0.001). In Kaplan-Meier Plotter survival analysis. **(F)** The breast cancer patients with higher expression of CHAF1A showed poor overall survival (OS) compared to patients with lower CHAF1A expression. **(G)** The breast cancer patients with higher expression of CHAF1A took less time to develop disease recurrence. **(H)** The breast cancer patients with higher CHAF1A expression took shorter time to develop cancer distant metastasis.

To further investigate the clinical significance and application of CHAF1A in breast cancer, the correlation between the expression of CHAF1A and breast cancer phenotype in UALCAN database was analyzed (Chandrashekar et al., 2017; Chen et al., 2019; Chen et al., 2022). Both mRNA expression and protein expression of CHAF1A were significantly elevated in breast cancer samples compared with normal samples (Figures 2A, B). Furthermore, among the subtypes of breast cancer, the expression of CHAF1A was significantly higher in triple negative breast cancer (TNBC) compared to either luminal or HER2 positive breast cancers in both mRNA level and protein level (Figures 2C, D). In addition, the expression of CHAF1A was higher in TP53 mutant tissues compared to TP53 wild type tissues in breast cancer (Figure 2E).

Then, we determined the potential effect of elevated CHAF1A expression on patients’ outcome. Elevated expression of CHAF1A was significantly correlated with shorter overall survival (OS) (Figure 2F), shorter recurrence free survival (RFS) (Figure 2G), and shorter distant metastasis free survival (DMFS) (Figure 2H) of breast cancer patients obtained from Kaplan-Meier plotter (Győrffy, 2021) survival analysis. The HR was 1.33, 1.25, and 1.48, respectively. As such, the data suggest that elevated expression of CHAF1A is a prognostic biomarker of poor patients’ outcome in breast cancer. The mechanisms underlying these events need to be further investigated.

### Co-expression gene network of CHAF1A and its potential mechanisms

Co-expression gene networks currently have been frequently used for exploration for the functional roles of the target genes (Villa-Vialaneix et al., 2013). In cBioPortal database (Cerami et al., 2012; Gao et al., 2013), the list of co-expression genes of CHAF1A in breast invasive carcinoma were downloaded, including three cohorts of TCGA data: Nature 2021, Cell 2015, and Firehose Legacy (Network, 2012; Ciriello et al., 2015). In order to get more precision co-expression networks, the genes shown consistent positive correlation score and consistent negative correlation score in all three cohorts were selected for further investigation.

**FIGURE 3:**
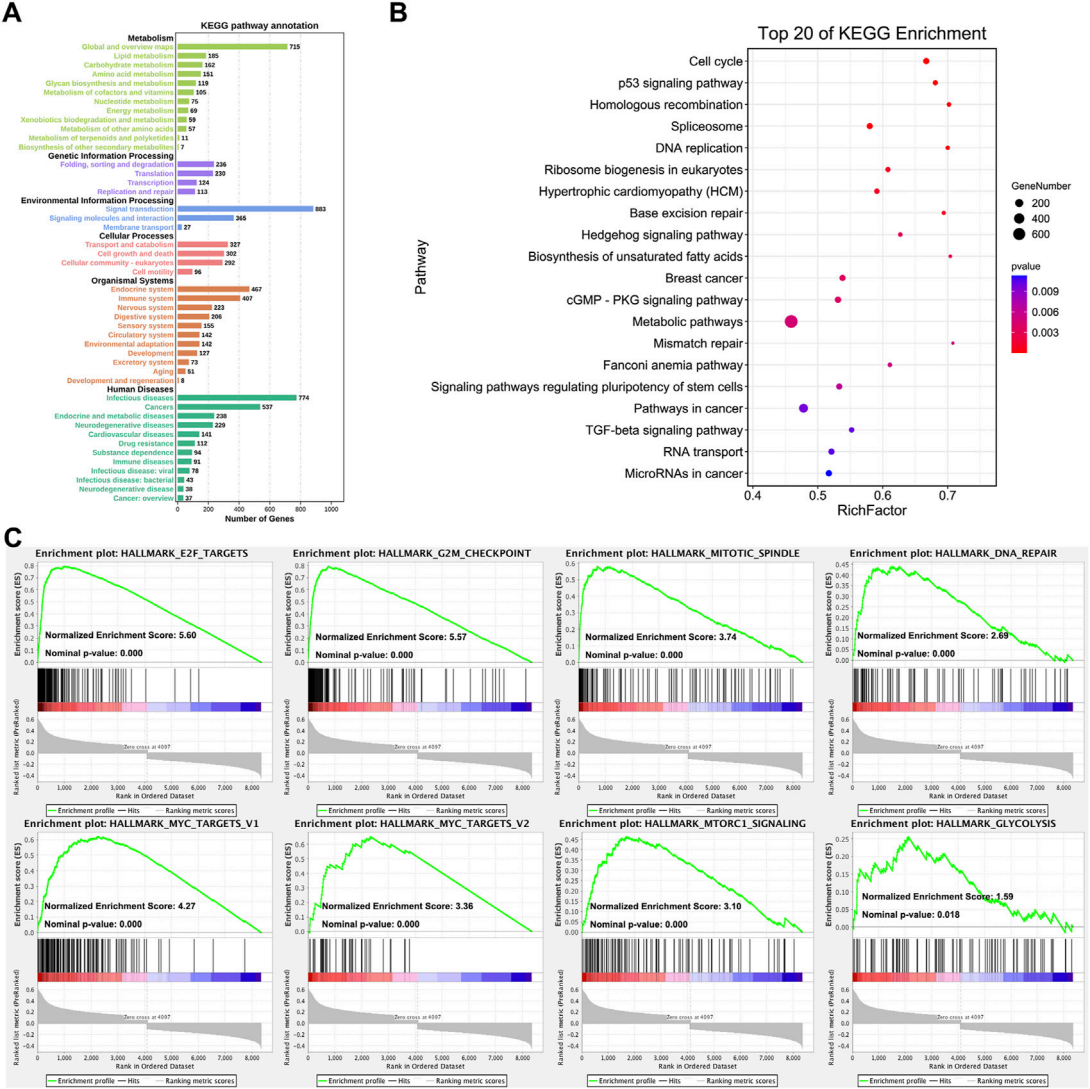
Analysis of CHAF1A’s functional roles and potential mechanisms. **(A)** The KEGG annotation of CHAF1A co-expression gene network was indicated by OmicShare. The length of the bar stands for the number of genes enriched in each function. **(B)** The top 20 pathways of CHAF1A co-expression gene network were generated by using OmicShare. The size of node stands for the exact number of genes that enriched in pathway, and the colour stands for *p*-value. **(C)** The enrichment of cancer hallmarks based on the CHAF1A co-expression gene network using GSEA.

The KEGG pathway annotation and enrichment of co-expression genes were generated with OmicShare online platform (Su et al., 2019; Wang et al., 2020). Figure 3 displayed the comprehensive functions and mechanisms of CHAF1A with statistical significance. The major functional roles of CHAF1A and its co-expressed genes focused on metabolism, genetic information processing, environmental information processing, cellular processes, organismal systems and human diseases (Figure 3A). Especially, the effect on metabolism (amino acid metabolism and energy metabolism), immune system, and drug resistance are more closely connected with potential response to cancer treatment. Among the top 20 KEGG pathway enrichment (Figure 3B), the co-expression network was covered cancer metabolism, stemness, microRNA, cell cycle, RNA splicing, TGF-β pathway, apoptosis and DNA repair.

In addition, the pathways involved in cancer hallmarks were investigated using GSEA (Mootha et al., 2003; Subramanian et al., 2005). As shown in Figure 3C, the co-expression network in TCGA breast cancer cohorts was significantly positively enriched in E2F targets pathway, G2M checkpoint pathway, mitotic spindle pathway, DNA repair pathway, MYC target pathway, MTORC1 signaling pathway, and glycolysis pathway. The results from GSEA analysis were specific to signaling pathways, while still consistent with that from OmicShare KEGG pathway annotation and enrichment. Interestingly, cancer metabolism was of the most outstanding generated by various analyses, which suggested that CHAF1A might be critical to breast cancer development and progression, thus might provide novel therapeutic target for the treatment of breast cancer.

### Association of elevated CHAF1A expression with immunosuppressive tumor microenvironment

**FIGURE 4:**
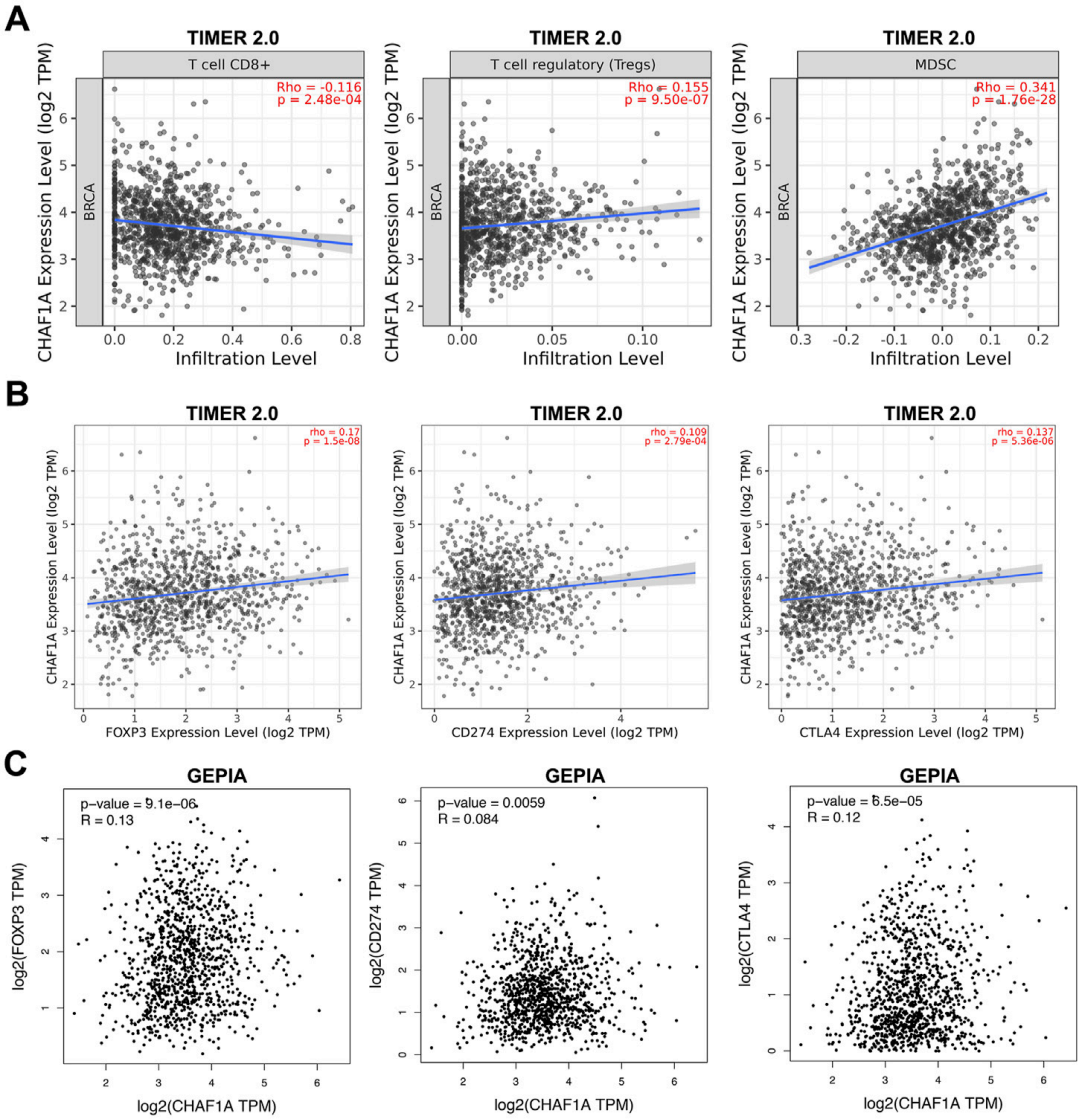
The correlation of CHAF1A with immune infiltrating cells and with immune checkpoint markers in breast cancer tissues. **(A)** Higher expression of CHAF1A was negatively associated with cancer infiltrating CD8^+^ T cells, but positively associated with infiltrating regulatory T cells and MDSC cells in breast cancer as indicated in TIMER 2.0 database. **(B)** The expression of CHAF1A was positively correlated with the expression of CD274 and CTLA4 in breast cancer in TIMER 2.0 database. **(C)** The expression of CHAF1A was positively correlated with the expression of CD274 and CTLA4 in breast cancer in GEPIA database.

Since the co-expression network analysis indicated that immune system might be involved (Figure 3A), we looked deep into the tumor-infiltrating immune cells of breast cancer samples to investigate the correlation between CHAF1A and immune response in TIMER 2.0 database (Li et al., 2016; Li et al., 2017; Li et al., 2020). In breast cancer, it was found that the expression of CHAF1A was negatively correlated with CD8^+^ T cell, but positively correlated with regulatory T cell (Treg) and MDSC (Myeloid-derived suppressor cell) (Figure 4A).

Furthermore, we checked the correlation between CHAF1A and immune checkpoints in TIMER 2.0 database. The expression of CHAF1A was positively correlated with that of both CD274 and CTLA4 (Figure 4B). This correlation between CHAF1A and either CD274 or CTLA4 was also confirmed by GEPIA database (Figure 4C). Thereby, the elevated expression of CHAF1A might be related with suppressive tumor microenvironment resulting in poor survival outcome. However, these patients might response to immunotherapy.

### Elevated CHAF1A expression used as a promising predictive biomarker to therapeutic treatment

In order to explore whether elevated CHAF1A expression detected in breast cancer patients’ samples could be used to predict the sensitivity of the patients to therapeutic treatment, the correlations between the oncolytic response of patients and elevated CHAF1A expression were determined in Kaplan-Meier plotter (Győrffy, 2021).

**FIGURE 5:**
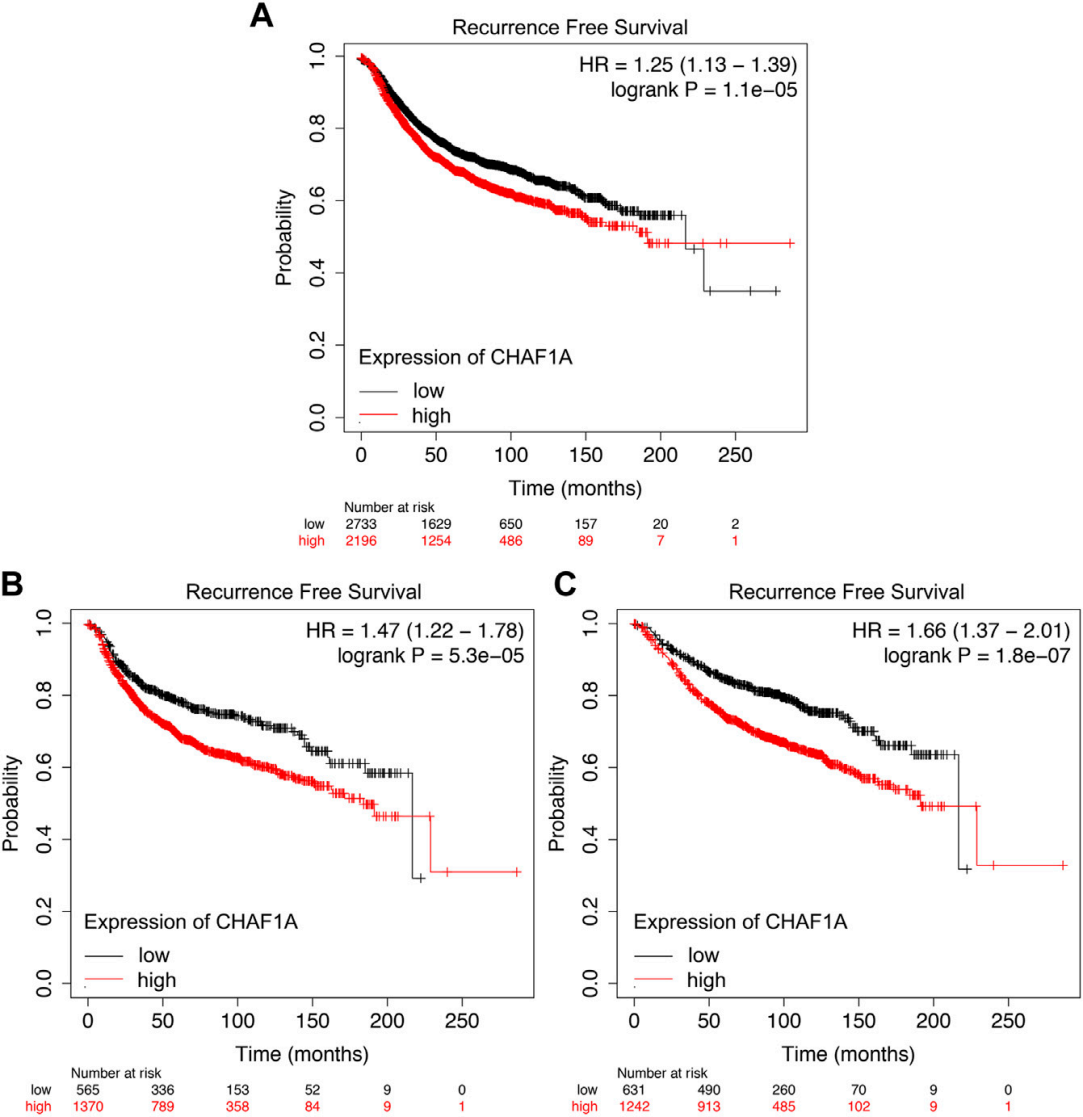
Resistance to endocrine therapy and chemotherapy treatment of breast cancer patients correlated with elevated CHAF1A expression as found by Kaplan-Meier Plotter. Breast cancer patients with elevated expression of CHAF1A showed shorter time to develop disease recurrence when they received **(A)** chemotherapy and endocrine therapy, **(B)** chemotherapy treatment, and **(C)** endocrine therapy treatment as found by Kaplan-Meier Plotter analysis.

It was shown that the breast cancer patients with elevated expression of CHAF1A showed significantly shorter recurrence free survival whether receiving endocrine therapy or chemotherapy (Figure 5A). Since the mechanisms of endocrine therapy and chemotherapy are different, the breast cancer patients were divided into two different groups, one group received endocrine therapy and the other one received chemotherapy. Patients with elevated expression of CHAF1A showed significantly shorter recurrence free survival in each group (Figures 5B,C). As such, breast cancer patients with higher expression of CHAF1A might progress into treatment resistance to endocrine therapy and chemotherapy in shorter time.

### CHAF1A highly expressed in human breast cancer tissues and its potential function

**FIGURE 6:**
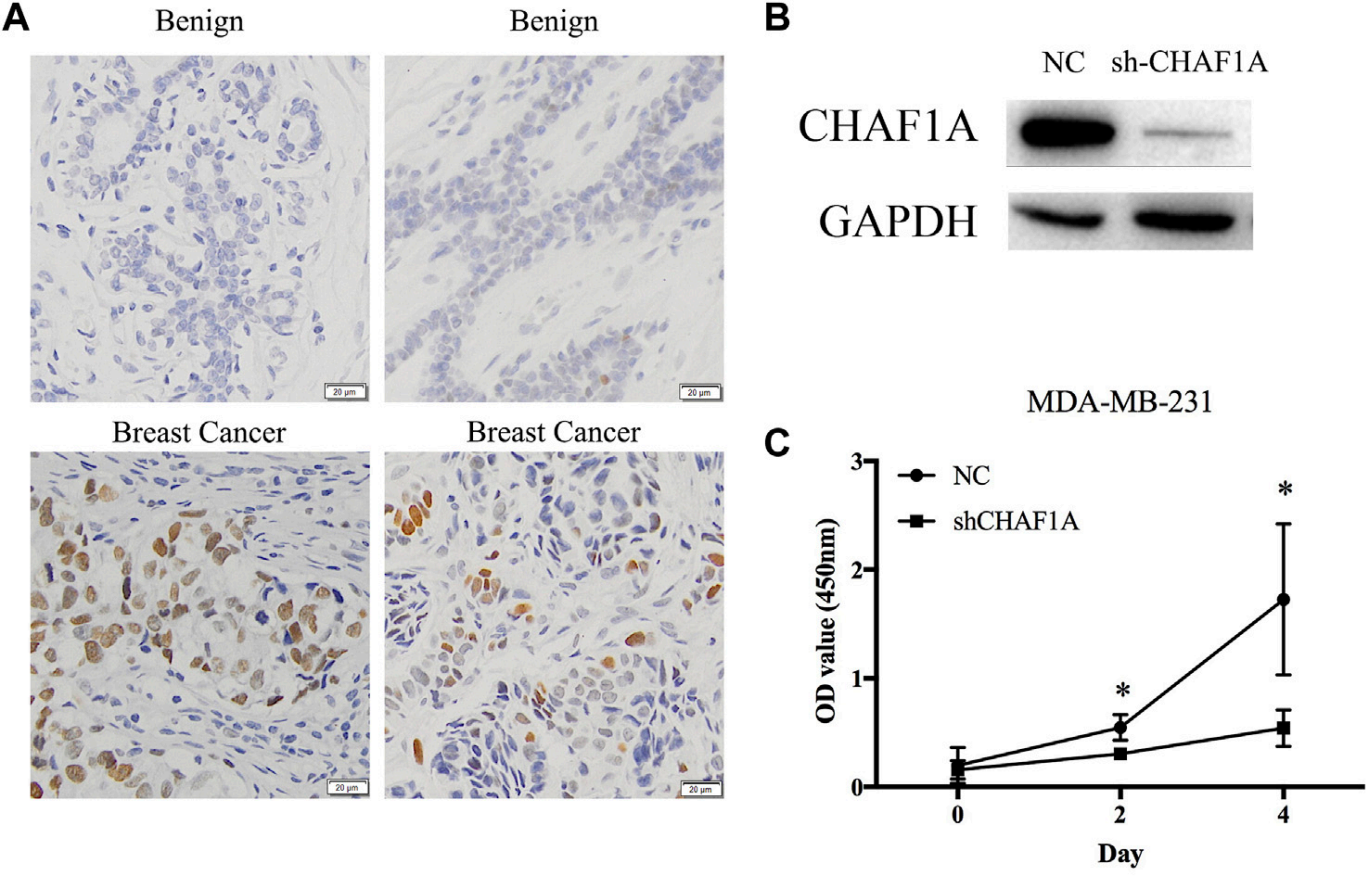
CHAF1A highly expressed in breast cancer tissues and inhibition of cell proliferation by silencing of CHAF1A. **(A)** Higher expression of CHAF1A was confirmed in clinical breast cancer tissues compared with normal tissues by IHC. **(B)** The expression of CHAF1A was dramatically downregulated using shRNA of CHAF1A in MDA-MB-231 cells. **(C)** Cell proliferation of MDA-MB-231 cells was significantly inhibited after CHAF1A silencing.

Clinical tissues were used to validate the expression of CHAF1A in patients. The expression of CHAF1A is much higher in breast cancer tissues compared with benign ones as shown by the IHC images (Figure 6A). To further investigate the function of CHAF1A in breast cancer, shRNA targeting CHAF1A was used to treat MDA-MB-231 cells. The expression of CHAF1A was significantly downregulated as shown in Figure 6B. Moreover, cell proliferation was inhibited when CHAF1A was silencing in MDA-MB-231 cells (Figure 6C), indicating that CHAF1A played important role in breast cancer cell growth. As such, CHAF1A may be a potential therapeutic target of breast cancer.

## Discussion

During the past decades, therapeutic options in cancers have been fast developed. The current treatment paradigm is now focused on mechanism-based therapeutics with selectivity, such as targeted therapy and immunotherapy (Vanneman and Dranoff, 2012). However, neither targeted therapy or immunotherapy can reach effective and durable results. Increasing evidences indicate that some targeted therapy can promote the anti-cancer immune response. As such, the combined use of both targeted therapy and immunotherapy may generate synergistic anti-cancer efficacy for patients (Bergholz et al., 2020). Thereby, the screening of immune-related therapeutic target is of great interests in cancer.

In this study, we have reported CHAF1A was overexpressed in 20 types of cancers. To rule out the potential effect of genomic alterations of CHAF1A in cancers, mutation, insertion, deletion and copy number alterations were investigated in COSMIC and Oncomine. The frequency of genetic alterations was too low to lead cancers development and progression. Elevated expression of CHAF1A has been reported to be associated with several solid cancers (Xu et al., 2016; Liu et al., 2017; Xia et al., 2017; Zheng et al., 2018; Tao et al., 2021), it was found that this phenomenon was commonly shared in more cancer types, indicating that abnormal higher expression of CHAF1A could be a potential biomarker for cancer diagnosis. Moreover, there was no study about the functional roles and mechanisms of CHAF1A in breast cancer. Thereby, we have shown the comprehensive profile of CHAF1A in breast cancer.

As a hormone related cancer type, the subtype of breast cancer classification is based on the expression of three major factors: estrogen receptor (ER), progesterone receptor (PR) and human epidermal growth factor receptor 2 (HER2). TNBC is typically characterized by lack of expression of ER, PR and HER2 (Garrido-Castro et al., 2019). More importantly, TNBC is the most aggressive subtype of breast cancer (Ensenyat-Mendez et al., 2021). Overexpression of CHAF1A was found in breast cancer tissues compared to benign tissues. This was confirmed in IHC analysis by using clinical breast cancer tissues compared with normal ones. Furthermore, the expression of CHAF1A was significantly higher in TNBC than either luminal or HER2 positive breast cancer. In addition, it was shown that the expression of CHAF1A was much higher in TP53 mutant breast cancer patients. Since it was reported that the frequency of TP53 mutation was high in TNBC (Network, 2012), it was consistent with our finding that the expression of CHAF1A was not only higher in TP53 mutant breast cancer, but also higher in TNBC. In addition, we demonstrated statistically significant correlation between elevated CHAF1A expression and poor breast cancer patients’ outcome. The results suggest that the elevated expression of CHAF1A may serve as a poor prognostic biomarker in breast cancer. Furthermore, silencing of CHAF1A can significantly inhibit MDA-MB-231 cell proliferation, which suggests that CHAF1A can serve as potential therapeutic target of breast cancer.

To identify the mechanisms of overexpression of CHAF1A in breast cancer, the bioinformatics analysis was applied using CHAF1A co-expression network. The major biological effects and signaling pathways demonstrated that CHAF1A played important roles in breast cancer progression. Some of these functions and mechanisms of CHAF1A were reported in other cancer types, however, most of these were shown in the first time. In ovarian cancer, it was found that CHAF1A was involved in DNA repair, apoptosis, and cell cycle (Xia et al., 2017), which were confirmed in our study by pathway enrichment as shown in KEGG pathways (such as cell cycle pathway, p53 signaling pathway, DNA replication pathway and mismatch repair pathway) and GSEA enrichment (such as E2F targets pathway, G2M checkpoint pathway, mitotic spindle pathway and DNA repair pathway). It was reported that CHAF1A could bind to the DNA promoter region of c-Myc to enhance the transcriptional expression of c-Myc in gastric cancer (Zheng et al., 2018), which was consistent with the current finding that CHAF1A was positively correlated with Myc targets. These findings indicated that CHAF1A played essential role to promote breast cancer growth. As such, CHAF1A could be potential therapeutic target in breast cancer.

More importantly, it was found that CHAF1A could affect cancer metabolism and immune system. In recent years, it has been demonstrated that cancer metabolism was important since it can affect immunotherapy and chemotherapy in cancer treatment (Zaal and Berkers, 2018; Bader et al., 2020; Desbats et al., 2020; DePeaux and Delgoffe, 2021). It was interesting to find that the elevated expression of CHAF1A was negatively correlated with CD8^+^ cell, but positively correlated with Treg and MDSC in breast cancer. Furthermore, we found that elevated expression of CHAF1A is positively associated with glycolysis in breast cancer. Since it is widely acknowledged that glycolysis is important factor to induce immunosuppressive microenvironment in cancer (Reinfeld et al., 2022). Thereby, it indicated that elevated CHAF1A might contribute to activation of glycolysis, which induced the immunosuppressive microenvironment. As well, the expression of CHAF1A was positively correlated with immune checkpoints, i.e., CD274 and CTLA4. As such, breast cancer patients with higher expression of CHAF1A might benefit from immune checkpoint inhibitors. And CHAF1A targeting therapy combined with immune checkpoint inhibitors might achieve synergistic effect for breast cancer patients with increased expression of CHAF1A.

Cancer metabolism reprogramming has been found to mediate drug resistance in patients (Chen et al., 2020). To further analyze the correlation between CHAF1A and the response to treatment, breast cancer patients received either endocrine therapy or chemotherapy were enrolled in Kaplan-Meier plotter. Since we found that CHAF1A could induce metabolic reprogramming in breast cancer, it was not surprising that patients with increased CHAF1A expression developed drug resistance in a relatively short period of time. This was consistent with the research finding that elevated expression of CHAF1A could promote thymidylate synthetase activity, leading to 5-FU resistance in gastric cancer (Wang et al., 2019). In addition, it was reported that abnormally elevated expression of CHAF1A could regulate the metabolic pathways of some amino acids, such as methionine, eventually inducing 5′-methylthioadenosine (MTA) accumulation in neuroblastoma (Tao et al., 2021). Homozygous deletion of the methylthioadenosine phosphorylase (MTAP) is frequently found in some types of cancer, and the application of purine analogue has been shown to be effective therapeutic option in MTAP deletion cancer patients (Tang et al., 2018). Since elevated expression of CHAF1A may cause accumulation of MTA *via* regulating amino acid metabolism, it is possible that purine analogue might be potential treatment option in MTAP deletion breast cancer patient with higher expression of CHAF1A.

The current work mainly investigated the bioinformatic analysis of CHAF1A. Thereby, further functional study of CHAF1A would better validate its potential role as biomarker and target in breast cancer. That is also the limitation of this study.

In general, the current study demonstrated that elevated expression of CHAF1A can be used as diagnostic biomarker in various types of human cancers. Moreover, elevated expression of CHAF1A is a promising prognostic predictor and potential biomarker of drug resistance in breast cancer. In addition, it may serve as a promising therapeutic target and biomarker to predict the sensitivity of immunotherapy in breast cancer patients.

## Data Availability

The original contributions presented in the study are included in the article/Supplementary Material, further inquiries can be directed to the corresponding author.

## References

[B1] Bader J. E. Voss K. Rathmell J. C. (2020). Targeting metabolism to improve the tumor microenvironment for cancer immunotherapy. Mol. Cell 78 (6), 1019–1033. 10.1016/j.molcel.2020.05.034 32559423 PMC7339967

[B2] Barbieri E. De Preter K. Capasso M. Chen Z. Hsu D. M. Tonini G. P. (2014). Histone chaperone CHAF1A inhibits differentiation and promotes aggressive neuroblastoma. Cancer Res. 74 (3), 765–774. 10.1158/0008-5472.CAN-13-1315 24335960

[B3] Bergholz J. S. Wang Q. Kabraji S. Zhao J. J. (2020). Integrating immunotherapy and targeted therapy in cancer treatment: Mechanistic insights and clinical implications. Clin. Cancer Res. 26 (21), 5557–5566. 10.1158/1078-0432.CCR-19-2300 32576627 PMC7641965

[B4] Cerami E. Gao J. Dogrusoz U. Gross B. E. Sumer S. O. Aksoy B. A. (2012). The cBio cancer genomics portal: an open platform for exploring multidimensional cancer genomics data. Cancer Discov. 2 (5), 401–404. 10.1158/2159-8290.CD-12-0095 22588877 PMC3956037

[B5] Chandrashekar D. S. Bashel B. Balasubramanya S. A. H. Creighton C. J. Ponce-Rodriguez I. Chakravarthi B. V. S. K. (2017). UALCAN: A portal for facilitating tumor subgroup gene expression and survival analyses. Neoplasia 19 (8), 649–658. 10.1016/j.neo.2017.05.002 28732212 PMC5516091

[B6] Chen F. Chandrashekar D. S. Varambally S. Creighton C. J. (2019). Pan-cancer molecular subtypes revealed by mass-spectrometry-based proteomic characterization of more than 500 human cancers. Nat. Commun. 10 (1), 5679. 10.1038/s41467-019-13528-0 31831737 PMC6908580

[B7] Chen X. Chen S. Yu D. (2020). Metabolic reprogramming of chemoresistant cancer cells and the potential significance of metabolic regulation in the reversal of cancer chemoresistance. Metabolites 10 (7), 289. 10.3390/metabo10070289 32708822 PMC7408410

[B8] Chen F. Chandrashekar D. S. Scheurer M. E. Varambally S. Creighton C. J. (2022). Global molecular alterations involving recurrence or progression of pediatric brain tumors. Neoplasia 24 (1), 22–33. 10.1016/j.neo.2021.11.014 34864569 PMC8649620

[B9] Ciriello G. Gatza M. L. Beck A. H. Wilkerson M. D. Rhie S. K. Pastore A. (2015). Comprehensive molecular portraits of invasive lobular breast cancer. Cell 163 (2), 506–519. 10.1016/j.cell.2015.09.033 26451490 PMC4603750

[B10] DePeaux K. Delgoffe G. M. (2021). Metabolic barriers to cancer immunotherapy. Nat. Rev. Immunol. 21 (12), 785–797. 10.1038/s41577-021-00541-y 33927375 PMC8553800

[B11] Desbats M. A. Giacomini I. Prayer-Galetti T. Montopoli M. (2020). Metabolic plasticity in chemotherapy resistance. Front. Oncol. 10, 281. 10.3389/fonc.2020.00281 32211323 PMC7068907

[B12] Ensenyat-Mendez M. Llinàs-Arias P. Orozco J. I. J. Íñiguez-Muñoz S. Salomon M. P. Sesé B. (2021). Current triple-negative breast cancer subtypes: Dissecting the most aggressive form of breast cancer. Front. Oncol. 11, 681476. 10.3389/fonc.2021.681476 34221999 PMC8242253

[B13] Gao J. Aksoy B. A. Dogrusoz U. Dresdner G. Gross B. Sumer S. O. (2013). Integrative analysis of complex cancer genomics and clinical profiles using the cBioPortal. Sci. Signal 6 (269), pl1. 10.1126/scisignal.2004088 23550210 PMC4160307

[B14] Garrido-Castro A. C. Lin N. U. Polyak K. (2019). Insights into molecular classifications of triple-negative breast cancer: Improving patient selection for treatment. Cancer Discov. 9 (2), 176–198. 10.1158/2159-8290.CD-18-1177 30679171 PMC6387871

[B15] Győrffy B. (2021). Survival analysis across the entire transcriptome identifies biomarkers with the highest prognostic power in breast cancer. Comput. Struct. Biotechnol. J. 19, 4101–4109. 10.1016/j.csbj.2021.07.014 34527184 PMC8339292

[B16] Houlard M. Berlivet S. Probst A. V. Quivy J. P. Héry P. Almouzni G. (2006). CAF-1 is essential for heterochromatin organization in pluripotent embryonic cells. PLoS Genet. 2 (11), e181. 10.1371/journal.pgen.0020181 17083276 PMC1630711

[B17] Lánczky A. Győrffy B. (2021). Web-based survival analysis tool tailored for medical research (KMplot): Development and implementation. J. Med. Internet Res. 23 (7), e27633. 10.2196/27633 34309564 PMC8367126

[B18] Li B. Severson E. Pignon J. C. Zhao H. Li T. Novak J. (2016). Comprehensive analyses of tumor immunity: Implications for cancer immunotherapy. Genome Biol. 17 (1), 174. 10.1186/s13059-016-1028-7 27549193 PMC4993001

[B19] Li T. Fan J. Wang B. Traugh N. Chen Q. Liu J. S. (2017). TIMER: A web server for comprehensive analysis of tumor-infiltrating immune cells. Cancer Res. 77 (21), e108–e110. 10.1158/0008-5472.CAN-17-0307 29092952 PMC6042652

[B20] Li T. Fu J. Zeng Z. Cohen D. Li J. Chen Q. (2020). TIMER2.0 for analysis of tumor-infiltrating immune cells. Nucleic Acids Res. 48 (W1), W509–W514. 10.1093/nar/gkaa407 32442275 PMC7319575

[B21] Liu T. Wei J. Jiang C. Wang C. Zhang X. Du Y. (2017). CHAF1A, the largest subunit of the chromatin assembly factor 1 complex, regulates the growth of H1299 human non-small cell lung cancer cells by inducing G0/G1 cell cycle arrest. Exp. Ther. Med. 14 (5), 4681–4686. 10.3892/etm.2017.5201 29201167 PMC5704333

[B22] Mootha V. K. Lindgren C. M. Eriksson K. F. Subramanian A. Sihag S. Lehar J. (2003). PGC-1alpha-responsive genes involved in oxidative phosphorylation are coordinately downregulated in human diabetes. Nat. Genet. 34 (3), 267–273. 10.1038/ng1180 12808457

[B23] Murzina N. Verreault A. Laue E. Stillman B. (1999). Heterochromatin dynamics in mouse cells: Interaction between chromatin assembly factor 1 and HP1 proteins. Mol. Cell 4 (4), 529–540. 10.1016/s1097-2765(00)80204-x 10549285

[B24] Network C. G. A. (2012). Comprehensive molecular portraits of human breast tumours. Nature 490 (7418), 61–70. 10.1038/nature11412 23000897 PMC3465532

[B25] Polo S. E. Theocharis S. E. Klijanienko J. Savignoni A. Asselain B. Vielh P. (2004). Chromatin assembly factor-1, a marker of clinical value to distinguish quiescent from proliferating cells. Cancer Res. 64 (7), 2371–2381. 10.1158/0008-5472.can-03-2893 15059888

[B26] Qin J. Sun X. Ma Y. Cheng Y. Ma Q. Jing W. (2021). Design, synthesis and biological evaluation of novel 1,3,4,9-tetrahydropyrano[3,4-b]indoles as potential treatment of triple negative breast cancer by suppressing PI3K/AKT/mTOR pathway. Bioorg Med. Chem. 55, 116594. 10.1016/j.bmc.2021.116594 34990979

[B27] Qu S. Ci X. Xue H. Dong X. Hao J. Lin D. (2018). Treatment with docetaxel in combination with Aneustat leads to potent inhibition of metastasis in a patient-derived xenograft model of advanced prostate cancer. Br. J. Cancer 118 (6), 802–812. 10.1038/bjc.2017.474 29381682 PMC5877435

[B28] Reinfeld B. I. Rathmell W. K. Kim T. K. Rathmell J. C. (2022). The therapeutic implications of immunosuppressive tumor aerobic glycolysis. Cell Mol. Immunol. 19 (1), 46–58. 10.1038/s41423-021-00727-3 34239083 PMC8752729

[B29] Rhodes D. R. Kalyana-Sundaram S. Mahavisno V. Varambally R. Yu J. Briggs B. B. (2007). Oncomine 3.0: Genes, pathways, and networks in a collection of 18,000 cancer gene expression profiles. Neoplasia 9 (2), 166–180. 10.1593/neo.07112 17356713 PMC1813932

[B30] Shen J. Liu X. Zhou M. Liu H. Xu L. Meng X. (2020). CHAF1A overexpression in human retinoblastoma promotes cell proliferation and suppresses apoptosis. J. BUON 25 (5), 2510–2514. 33277876

[B31] Su M. Guo C. Liu M. Liang X. Yang B. (2019). Therapeutic targets of vitamin C on liver injury and associated biological mechanisms: A study of network pharmacology. Int. Immunopharmacol. 66, 383–387. 10.1016/j.intimp.2018.11.048 30530052

[B32] Subramanian A. Tamayo P. Mootha V. K. Mukherjee S. Ebert B. L. Gillette M. A. (2005). Gene set enrichment analysis: a knowledge-based approach for interpreting genome-wide expression profiles. Proc. Natl. Acad. Sci. U. S. A. 102 (43), 15545–15550. 10.1073/pnas.0506580102 16199517 PMC1239896

[B33] Sung H. Ferlay J. Siegel R. L. Laversanne M. Soerjomataram I. Jemal A. (2021). Global cancer statistics 2020: GLOBOCAN estimates of incidence and mortality worldwide for 36 cancers in 185 countries. CA Cancer J. Clin. 71, 209–249. 10.3322/caac.21660 33538338

[B34] Sykaras A. G. Pergaris A. Theocharis S. (2021). Challenging, accurate and feasible: CAF-1 as a tumour proliferation marker of diagnostic and prognostic value. Cancers (Basel) 13 (11), 2575. 10.3390/cancers13112575 34073937 PMC8197349

[B35] Tang Z. Li C. Kang B. Gao G. Zhang Z. (2017). GEPIA: a web server for cancer and normal gene expression profiling and interactive analyses. Nucleic Acids Res. 45 (W1), W98–W102. 10.1093/nar/gkx247 28407145 PMC5570223

[B36] Tang B. Lee H. O. An S. S. Cai K. Q. Kruger W. D. (2018). Specific targeting of *MTAP*-deleted tumors with a combination of 2'-fluoroadenine and 5'-methylthioadenosine. Cancer Res. 78 (15), 4386–4395. 10.1158/0008-5472.CAN-18-0814 29844120 PMC6072572

[B37] Tao L. Moreno-Smith M. Ibarra-García-Padilla R. Milazzo G. Drolet N. A. Hernandez B. E. (2021). CHAF1A blocks neuronal differentiation and promotes neuroblastoma oncogenesis via metabolic reprogramming. Adv. Sci. (Weinh) 8 (19), e2005047. 10.1002/advs.202005047 34365742 PMC8498874

[B38] Tate J. G. Bamford S. Jubb H. C. Sondka Z. Beare D. M. Bindal N. (2019). COSMIC: the catalogue of somatic mutations in cancer. Nucleic Acids Res. 47 (D1), D941–D947. 10.1093/nar/gky1015 30371878 PMC6323903

[B39] Vanneman M. Dranoff G. (2012). Combining immunotherapy and targeted therapies in cancer treatment. Nat. Rev. Cancer 12 (4), 237–251. 10.1038/nrc3237 22437869 PMC3967236

[B40] Villa-Vialaneix N. Liaubet L. Laurent T. Cherel P. Gamot A. SanCristobal M. (2013). The structure of a gene co-expression network reveals biological functions underlying eQTLs. PLoS One 8 (4), e60045. 10.1371/journal.pone.0060045 23577081 PMC3618335

[B41] Wang D. Li X. Shen B. Chen X. Shu Y. (2019). Histone chaperone CHAF1A impacts the outcome of fluoropyrimidines-based adjuvant therapy in gastric cancer by regulating the expression of thymidylate synthetase. Gene 716, 144034. 10.1016/j.gene.2019.144034 31377317

[B42] Wang S. Su W. Zhong C. Yang T. Chen W. Chen G. (2020). An eight-CircRNA assessment model for predicting biochemical recurrence in prostate cancer. Front. Cell Dev. Biol. 8, 599494. 10.3389/fcell.2020.599494 33363156 PMC7758402

[B43] Xia D. Yang X. Liu W. Shen F. Pan J. Lin Y. (2017). Over-expression of CHAF1A in Epithelial Ovarian Cancer can promote cell proliferation and inhibit cell apoptosis. Biochem. Biophys. Res. Commun. 486 (1), 191–197. 10.1016/j.bbrc.2017.03.026 28286267

[B44] Xu M. Jia Y. Liu Z. Ding L. Tian R. Gu H. (2016). Chromatin assembly factor 1, subunit A (P150) facilitates cell proliferation in human hepatocellular carcinoma. Onco Targets Ther. 9, 4023–4035. 10.2147/OTT.S107050 27445493 PMC4936808

[B45] Zaal E. A. Berkers C. R. (2018). The influence of metabolism on drug response in cancer. Front. Oncol. 8, 500. 10.3389/fonc.2018.00500 30456204 PMC6230982

[B46] Zheng L. Liang X. Li S. Li T. Shang W. Ma L. (2018). CHAF1A interacts with TCF4 to promote gastric carcinogenesis via upregulation of c-MYC and CCND1 expression. EBioMedicine 38, 69–78. 10.1016/j.ebiom.2018.11.009 30449701 PMC6306399

