# Supplementary material for: Overexpression of CHAF1A is associated with poor prognosis, tumor immunosuppressive microenvironment and treatment resistance
Source: Front Genet. 2023 Mar 9;14:1108004. doi: 10.3389/fgene.2023.1108004 (PMC10033519; doi:10.3389/fgene.2023.1108004)
Supplement: Supplementary file 1 [file Table1.DOCX]

**Supplementary Table 1. Copy number alterations of *CHAF1A* in human cancers (COSMIC database)**

| Cancer Type | Copy Number Gain | Copy Number Loss | Total Number of Cancers |
| --- | --- | --- | --- |
| Breast | 0 | 2 (0.134%) | 1492 |
| Central nervous system | 1 (0.097%) | 0 | 1035 |
| Endometrium | 0 | 2 (0.341%) | 586 |
| Haematopoietic and lymphoid | 1 (0.151%) | 0 | 661 |
| Kidney | 0 | 1 (0.101%) | 995 |
| Liver | 0 | 2 (0.302%) | 663 |
| Lung | 1 (0.099%) | 0 | 1006 |
| Oesophagus | 0 | 2 (0.392%) | 510 |
| Ovary | 0 | 7 (1.023%) | 684 |
| Pancreas | 0 | 1 (0.111%) | 898 |
| Prostate | 0 | 1 (0.105%) | 949 |
| Skin | 0 | 3 (0.511%) | 587 |
| Stomach | 0 | 4 (0.847%) | 472 |
| Upper aerodigestive tract | 0 | 6 (1.154%) | 520 |
| Urinary tract | 0 | 1 (0.251%) | 399 |

**Supplementary Table 2. The expression of *CHAF1A* in Cancer Tissues vs Normal Tissues (Oncomine database)**

| Cancer type | Cohort name | Subtype of cancer | Number of cancer tissues | Number of normal tissues | Fold Change  (cancer vs normal) | *p* value |
| --- | --- | --- | --- | --- | --- | --- |
| Bladder Cancer | Sanchez-Carbayo Bladder 2 | Superficial Bladder Cancer | 28 | 48 | 3.296 | 2.05E-19 |
|  |  | Infiltrating Bladder Urothelial Carcinoma | 81 | 48 | 1.935 | 2.93E-9 |
|  | Dyrskjot Bladder 3 | Superficial Bladder Cancer | 28 | 14 | 1.691 | 1.65E-8 |
|  | Blaveri Bladder 2 | Superficial Bladder Cancer | 26 | 3 | 2.295 | 0.003 |
| Breast Cancer | TCGA Breast | Mixed Lobular and Ductal Breast Carcinoma | 7 | 61 | 1.502 | 1.05E-5 |
|  |  | Invasive Ductal Breast Carcinoma | 389 | 61 | 1.800 | 5.11E-34 |
|  |  | Invasive Breast Carcinoma | 76 | 61 | 1.756 | 1.56E-17 |
|  | Richardson Breast 2 | Ductal Breast Carcinoma | 40 | 7 | 1.792 | 9.93E-8 |
| Cervical Cancer | Scotto Cervix 2 | Cervical Squamous Cell Carcinoma | 32 | 24 | 8.434 | 1.22E-16 |
|  | Zhai Cervix | Cervical Squamous Cell Carcinoma Epithelia | 21 | 10 | 1.933 | 6.64E-6 |
| Colorectal Cancer | Gaedcke Colorectal | Rectal Adenocarcinoma | 65 | 65 | 1.998 | 3.56E-23 |
| Esophageal Cancer | Kimchi Esophagus | Esophageal Adenocarcinoma | 8 | 8 | 5.439 | 6.21E-4 |
|  | Hu Esophagus | Esophageal Squamous Cell Carcinoma | 17 | 17 | 1.585 | 3.41E-6 |
| Head and Neck Cancer | Schlingemann Head-Neck | Hypopharyngeal Squamous Cell Carcinoma | 4 | 4 | 1.688 | 0.029 |
| Kidney Cancer | Cutcliffe Renal | Renal Wilms Tumor | 18 | 3 | 1.843 | 2.41E-4 |
| Leukemia | Andersson Leukemia | T-Cell Acute Lymphoblastic Leukemia | 11 | 6 | 3.227 | 4.13E-8 |
| Lung Cancer | Hou Lung | Squamous Cell Lung Carcinoma | 27 | 65 | 2.386 | 8.28E-17 |
|  |  | Large Cell Lung Carcinoma | 19 | 65 | 2.239 | 1.34E-7 |
|  | Garber Lung | Small Cell Lung Carcinoma | 4 | 6 | 4.694 | 7.42E-4 |
|  |  | Large Cell Lung Carcinoma | 4 | 6 | 3.128 | 0.003 |
|  | Bhattacharjee Lung | Squamous Cell Lung Carcinoma | 21 | 17 | 5.637 | 9.92E-5 |
| Lymphoma | Basso Lymphoma | Burkitt's Lymphoma | 17 | 25 | 2.975 | 8.87E-8 |
|  |  | Primary Effusion Lymphoma | 9 | 25 | 2.762 | 1.09E-5 |
| Ovarian Cancer | Yoshihara Ovarian | Ovarian Serous Adenocarcinoma | 43 | 10 | 2.975 | 1.14E-14 |
| Pancreatic Cancer | Grutzmann Pancreas | Pancreatic Ductal Adenocarcinoma Epithelia | 11 | 11 | 1.696 | 0.016 |
| Prostate Cancer | Tomlins Prostate | Benign Prostatic Hyperplasia Epithelia | 3 | 16 | 1.820 | 0.011 |
| Sarcoma | Quade Uterus | Leiomyosarcoma | 4 | 4 | 12.107 | 0.002 |
|  |  | Uterine Corpus Leiomyosarcoma | 9 | 4 | 4.347 | 0.002 |
|  | Detwiller Sarcoma | Pleomorphic Liposarcoma | 3 | 15 | 2.420 | 1.46E-4 |
|  |  | Fibrosarcoma | 7 | 15 | 2.721 | 3.93E-5 |
|  | Barretina Sarcoma | Pleomorphic Liposarcoma | 23 | 9 | 1.602 | 1.65E-7 |
|  |  | Myxofibrosarcoma | 31 | 9 | 1.805 | 1.44E-9 |
|  |  | Leiomyosarcoma | 26 | 9 | 1.781 | 1.59E-8 |
| Adrenal Cancer | Giordano Adrenal | Adrenal Cortex Carcinoma | 9 | 3 | 3.339 | 9.63E-5 |
| Vulva Cancer | Santegoets Vulva | Vulvar Intraepithelial Neoplasia | 9 | 10 | 2.393 | 2.48E-7 |
| Skin Cancer | Nindl Skin | Skin Squamous Cell Carcinoma | 5 | 6 | 1.728 | 0.007 |
